# GABAergic signalling in the suprachiasmatic nucleus is required for coherent circadian rhythmicity

**DOI:** 10.1111/ejn.16582

**Published:** 2024-11-18

**Authors:** Nathan Klett, Heinrich S. Gompf, Charles N. Allen, Olga Cravetchi, Lauren M. Hablitz, Ali N. Gunesch, Robert P. Irwin, William D. Todd, Clifford B. Saper, Patrick M. Fuller

**Affiliations:** ^1^ Oregon Institute for Occupational Health Sciences USA; ^2^ Neuroscience Graduate Program USA; ^3^ Department of Neurological Surgery University of California, Davis Davis CA USA; ^4^ Department of Behavioral Neuroscience Oregon Health & Science University Portland OR USA; ^5^ Department of Neurology, Division of Sleep Medicine, and Program in Neuroscience Beth Israel Deaconess Medical Center, Harvard Medical School Boston MA USA; ^6^ Present address: Center for Translational Neuromedicine University of Rochester Medical Center Rochester NY USA; ^7^ Present address: Department of Zoology and Physiology University of Wyoming Laramie WY USA

**Keywords:** circadian rhythm, GABA, mouse, synaptic transmission, vesicular GABA transporter

## Abstract

The suprachiasmatic nucleus is the circadian pacemaker of the mammalian brain. Suprachiasmatic nucleus neurons display synchronization of their firing frequency on a circadian timescale, which is required for the pacemaker function of the suprachiasmatic nucleus. However, the mechanisms by which suprachiasmatic nucleus neurons remain synchronized in vivo are poorly understood, although synaptic communication is considered indispensable. Suprachiasmatic nucleus neurons contain the neurotransmitter GABA and express GABA receptors. This has inspired the hypothesis that GABA signalling may play a central role in network synchronization, although this remains untested in vivo. Here, using local genetic deletion, we show that disruption of GABA synaptic transmission within the suprachiasmatic nucleus of adult mice results in the eventual deterioration of physiological and behavioural rhythmicity in vivo and concomitant cellular desynchrony in vitro. These findings suggest that intercellular GABA signalling is essential for behavioural rhythmicity and cellular synchrony of the suprachiasmatic nucleus neural network.

## Graphical abstract

Genetic disruption of GABAergic synaptic transmission by deletion of the vesicular GABA transporter within the suprachiasmatic nucleus of adult mice results in the eventual deterioration of physiological and behavioural rhythmicity in vivo and concomitant neuronal desynchrony in vitro. These data demonstrate that intercellular GABA signalling within the suprachiasmatic nucleus neural network is crucial for circadian behavioural rhythmicity and cellular synchrony. Visual abstract created with BioRender.com

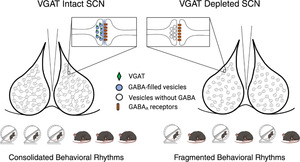

**Abbreviations**
AAV: adeno‐associated viral vector
ANOVA: analysis of variance
CCD: charge‐coupled device camera
CMV: cytomegalovirus
CNQX: cyanquixaline
DAB: 3,3‐diaminobenzidine tetrahydrochloride
DEPC: diethylprocarbonate
DIG: Digoxigenin
GABA: γ − aminobutyric acid
GABA_tonic_
: tonic GABA_A_ receptor‐mediated current
GEE: Generalized Estimating Equation
GFP: Green fluorescent protein
HBSS: Hanks' buffered salt solution
LD: light–dark
LMA: locomotor activity
mGPSCs: miniature GABAergic postsynaptic currents
NLS: nuclear localization sequence
IP: intraperitoneal
PBS: phosphate‐buffered saline
PER1: Period 1 protein
PER2: Period 2 protein
PER2::LUC: PER2::LUCIFERASE
RNA: Ribonucleic acid
SCN: suprachiasmatic nucleus
SPZ: subparaventricular zone
SSC: Standard saline citrate
Tb: body temperature
TBS: Tris‐buffered saline
TSA: Tyramide signal amplification
TTX: tetrodotoxin
VGAT: vesicular GABA transporter
VIP: vasoactive intestinal peptide
ZT: Zeitgeber Time

## 1 INTRODUCTION

The master mammalian circadian oscillator, located in the suprachiasmatic nucleus (SCN) of the hypothalamus, is composed of neurons that act as cell‐autonomous circadian oscillators (see Mohawk & Takahashi, 2011 for review). SCN neurons exhibit a wide range of periods and phase relationships when cultured at low density. In contrast, neurons are synchronized to a common period and phase in SCN explants or brain slices and remain synchronized for many cycles, highlighting the importance of the SCN interneural network for synchronization (Herzog et al., 1998; Welsh et al., 1995; Yamaguchi et al., 2003). Indeed, the SCN neural network can stabilize circadian rhythms, despite mutations to the molecular clock (Liu et al., 2007). The mechanisms by which SCN neurons remain synchronized in vivo are poorly understood, although synaptic communication within the SCN network is considered indispensable (Aton et al., 2006, 2005; Granados‐Fuentes et al., 2024; Liu et al., 2007; Liu & Reppert, 2000; Webb et al., 2009; Yamaguchi et al., 2003).

While SCN neurons differ in their neuropeptide content, virtually all SCN neurons synthesize GABA and express GABA receptors (Moore & Speh, 1993). In vitro studies using dispersed SCN neurons or slice cultures have, however, provided mixed results concerning whether local GABAergic transmission synchronizes the rhythms of individual SCN neurons (Albus et al., 2005; Aton et al., 2006; Freeman et al., 2013; Liu & Reppert, 2000). Daily application of GABA synchronizes the firing rate rhythm of individual SCN neurons maintained in dispersed cell cultures (Liu & Reppert, 2000). Yet the inhibition of GABA_A_ receptors does not affect the synchronization of individual cellular oscillators in cultured SCN explants (Aton et al., 2006). In contrast, genetic deletion of GABA_A_ receptor γ2 and δ subunits decreased the synchrony of SCN neurons (Granados‐Fuentes et al., 2024). Importantly, GABA transmission has been shown to regulate the synchrony between SCN subregions (Albus et al., 2005; Evans et al., 2013). A significant barrier to understanding the role of GABA in the SCN has been the lack of methods to produce long‐term modulation of the GABA system in vivo (Ono et al., 2018). For example, global knockout of the vesicular GABA transporter (VGAT), which is required for synaptic GABA release, is embryonically lethal (Wojcik et al., 2006). Furthermore, genetic disruption of the GABA_A_ receptor is not feasible due to the number of possible subunit combinations that can constitute a functional receptor (Sigel & Steinmann, 2012). Here, we used an intersectional genetics approach to delete *Vgat* locally within the adult mouse SCN. We subsequently used physiological and behavioural recordings, ex vivo monitoring of SCN PER2 bioluminescence and single‐cell SCN *Per1* imaging to demonstrate that deletion of *Vgat* in the SCN severely disrupts the circadian timing system.

## 2 MATERIALS AND METHODS

### 2.1 Animals

Adult male *Vgat* ^lox/lox^, *Vgat* ^lox/+^, *Vgat* ^*+/+*^, *Vgat* ^lox/lox^; PERIOD2::LUCIFERASE (PER2::LUC) and *Vgat* ^lox/lox^; *Per1*‐Venus and C57Bl/6 mice [8–11 weeks, 17–24 g; n = 83] were used in this study. *Vgat* ^lox/lox^ mice (*Slc32a1tm1Lowl*/J) and *R26‐loxSTOPlox‐L10‐GFP* reporter mice were provided by Qingchun Tong and Brad Lowell, *Per1*‐Venus mice were provided by Karl Obrietan and Mary Cheng, Ohio State University, OH, USA, and PER2::LUC mice (B6.129S6‐*Per2tm1Jt*/J) were obtained from Jackson Labs (006852). All procedures, including generating the hybrid lines, were performed in accordance with the guidelines of the Animal Care and Use Committees of Beth Israel Deaconess Medical Center and Oregon Health & Science University.

### 2.2 Surgery

Mice were anaesthetized with ketamine/xylazine [100 and 10 mg/kg respectively, intraperitoneal (IP)] and then placed in a stereotaxic apparatus. Bilateral injections of an adeno‐associated viral (AAV, serotype 10) vector expressing a Cre‐recombinase‐GFP fusion protein under a CMV promoter (AAV10‐CMV‐NLS‐CRE‐GFP; NLS = Nuclear Localization Sequence, henceforth referred to as **AAV‐Cre**, 4.×10^13^ VGC/ml; 10–30 nl) or GFP in isolation (**AAV‐GFP**; 10–30 nl) were placed into the SCN (AP = −0.45 mm, ML = ±0.1 mm, DV = −5.5 mm) of *Vgat* ^lox/lox^ and *Vgat* ^lox/lox^; PER2::LUC mice using a compressed air delivery system. For the subparaventricular zone (SPZ) experiments, bilateral injections of an AAV‐expressing both Cre and Venus (a modified GFP) via a 2A self‐cleaving peptide under a Synapsin promoter (AAV8‐Syn‐iCre‐2A‐Venus [**AAV‐Cre‐Venus**]; 9.4 × 10^12^ VGC/ml; 10–15 nl; plasmid was a kind gift from Dr. Rolf Sprengel) or GFP in isolation (AAV‐GFP; 10–15 nl) were placed into the SPZ (AP = −0.4 mm, ML = ±0.2 mm, DV = −5.1 mm) of *Vgat* ^lox/lox^ mice, also using a compressed air delivery system. For the experiments employing the *Vgat* ^lox/lox^; *Per1*‐Venus mice, bilateral injections of an AAV vector expressing a Cre‐recombinase‐mCherry fusion protein (**AAV8‐Cre‐mCherry**; 0.2 ul) were performed using a Nanoject microinjector (Drummond Scientific Co., Broomall, PA). Immediately following the brain injections, mice used in experiments recording body temperature (Tb) and locomotor activity (LMA) were implanted IP with biotelemetry transmitters (Data Sciences International, St. Paul, MN). All incisions were sutured and treated with a topical antibiotic, and all animals received flunixin as an analgesic for 48 hrs during recovery.

### 2.3 Tb and LMA recordings

Mice recovered from surgery for 2–3 weeks before Tb and LMA recording began. The animals were individually housed in standard plastic mouse cages with ad libitum food (Lab Diet, St. Louis, MO) and water. Each cage was placed on top of a telemetry receiver interfaced with a data acquisition system (Data Sciences International, New Brighton, MN, USA). Tb values were recorded at 5‐min intervals, and LMA data were collected in 5‐min bins. The cages were housed inside isolation chambers, which provided ventilation, an ambient temperature of 23 ± 1°C, and visual isolation. Cages were changed every other week, and health checks were performed daily by real‐time analysis of the telemetry data; thus, the mice were not disturbed during the daily health checks. A white‐light LED matrix provided ambient environmental lighting.

### 2.4 Analysis of Tb and LMA rhythms

Data were analysed and plotted using Clocklab (Coulbourne Instruments, Natick, MA). The phase angle of Tb entrainment (“Tb psi” in Tables 1 and 2) was calculated as the difference (in hours) between the time of the light–dark (LD) transition and the acrophase of the Tb rhythm. The acrophase of the Tb rhythm was calculated from a least squared fit during the last 7 days of LD. A two‐factor repeated measures ANOVA was used to compare rhythm period, mean and amplitude between lighting and genotype conditions. Specific mean comparisons were made using Tukey's HSD post hoc test (SPSS). An α < 0.01 was used for all tests. All data are reported as mean ± standard deviation.

**TABLE 1:** Light masking SCN telemetry recordings.

SCN groups	Tb amplitude (LD)	Tb psi (hours, LD)	Tb amplitude (DD)	Tb period (DD)
*Cre‐AAV SCN (n = 6 LD ‐ > DD)	0.43 ± 0.07	2.54 ± 0.45 (p < 0.001 v GFP)	N/A	Arrhythmic
*Cre‐AAV SCN (n = 5 direct to DD)	‐	‐	N/A	Arrhythmic
**GFP‐AAV SCN (n = 6 LD ‐ > DD)	0.51 ± 0.03	4.78 ± 0.21	0.54 ± 0.04	24.05 ± 0.05 (23.82–24.12 h)
**Cre‐AAV SCN (n = 4 LD ‐ > DD)	0.52 ± 0.09	4.91 ± 0.13	0.53 ± 0.1	23.79 ± 0.11 (23.65–23.95)

* Confirmed bilateral SCN *Vgat* deletion.

** Wildtype Cre‐AAV SCN injected mice.

**TABLE 2:** SPZ telemetry recordings.

SPZ groups	Tb amplitude (LD)	Tb psi (hours; LD)	Tb amplitude (DD)	Tb period (DD)
*Cre‐AAV SPZ (n = 9)	0.54 ± 0.3	4.68 ± 0.17	0.46 ± 0.02	23.95 ± 0.03
**GFP‐AAV SPZ (n = 9)	0.56 ± 0.2	4.96 ± 0.26	0.52 ± 0.02	23.97 ± 0.03

* Confirmed bilateral SPZ *Vgat* deletion.

** Littermates of Cre‐AAV SCN injected mice.

### 2.5 Isotopic in situ hybridization

*Vgat* expression was monitored using 35^S^‐labeled riboprobes. The plasmid was linearized and transcribed with T3 polymerases to produce antisense RNA probes that were 35^S^‐radiolabeled. Sections were mounted, and hybridization was performed as previously described (Gooley et al., 2001; Kong et al., 2012). Probes were visualized by dipping the slides in a photographic emulsion, exposing them for 2–4 weeks, then developing in Kodak D‐19, fixing, drying and cover‐slipping the slides.

### 2.6 Digoxigenin (DIG)‐labelled RNA probe in situ hybridization

Six weeks after SCN‐directed injections of AAV‐Cre‐GFP, the Cre‐injected mice and uninjected littermates were given an intracerebroventricular injection of colchicine and transcardially perfused 36 hours later. Brain sections were cut in RNAse‐free conditions at 30 μm, preserved in RNAlater® RNA Stabilization Solution and frozen until use. Sections were washed in RNase‐free PBS containing diethylpyrocarbonate (DEPC), then incubated in a hybridization buffer for 1 h at 53°C. *Vgat* probe was denatured at 80°C for 10 min and then incubated in the hybridization buffer overnight at 53°C. Sections were successively washed in 2X standard saline citrate (SSC) with 50% formamide solution and 3 × 1 h in 1X SSC 50% with formamide solution, both at 53°C. After tris buffered saline (TBS) pH 7.5 washes, sections were incubated in 1% blocking reagent (Roche Applied Science, Penzberg, Germany) for 30 min, then incubated overnight in peroxidase‐conjugated DIG antibody (1:500, Roche Applied Science, Penzberg, Germany). Following TBS washes, sections were reacted with Tyramide signal amplification (TSA) Cy3 (1:50, Perkin Elmer, Waltham, MA) for 30 min. The *Vgat* probe was a kind gift from Dr. Shigafumi Yokota, Shimane University, Izumo, Japan.

### 2.7 Immunohistochemistry

A total of 40 μm brain sections were washed in phosphate‐buffered saline (PBS) and incubated in primary Cre, or GFP antiserum diluted in PBS containing 0.3% Triton X‐100 and 0.2% sodium azide overnight at room temperature. The Cre antibody (1:5 K, Novagen #69050) was a rabbit polyclonal IgG antibody. The chicken polyclonal antibody against GFP was raised against GFP isolated directly from the jellyfish *Aequorea Victoria* (Invitrogen; catalog number A10262). This antibody did not stain anything in the brains of uninjected wild‐type mice. The vasoactive intestinal peptide (VIP) antibody was a rabbit polyclonal antibody raised against porcine VIP conjugated to bovine thyroglobulin (1:10 K, ImmunoStar, #20077). It stained a typical pattern of VIP neuronal cell bodies in the ventral SCN and axons in the SPZ. Following primary antibody incubation, sections were washed in PBS and incubated in biotinylated secondary antiserum (against appropriate species IgG, 1:1000) in PBS for one hour, washed in PBS and incubated in ABC reagents for 1 hour. Sections were then washed in PBS and incubated in a solution of 0.06% 3,3‐diaminobenzidine tetrahydrochloride (DAB, Sigma) and 0.02% H_2_O_2_. The primary antibodies were omitted for all secondary antibody immunohistochemical controls, and the tissue showed no immunoreactivity above the background.

### 2.8 In vitro electrophysiology

Mice were entrained to a 12:12 LD cycle, with lights on at 10 AM. Mice were removed from housing, anaesthetized with isoflurane and decapitated. The brain was quickly removed and submerged in an ice‐cold slicing solution consisting of (in mM): 126 NaCl, 26 NaHCO_3_, 11 dextrose, 4 MgCl_2_, 2.5 KCl, 1.2 NaH_2_PO_4_ and 0.5 CaCl_2_, saturated with 95% O2, 5% CO2, ~300 mOsm. The brain was blocked, and 250 μm thick coronal slices were prepared with a Leica VT1000S vibratome. Slices were then transferred to artificial cerebrospinal fluid containing (in mM): 132.5 NaCl, 22 NaHCO_3_, 11 dextrose, 2.5 KCl, 2.4 CaCl_2_, 1.2 MgCl_2_ and 1.2 NaH_2_PO_4_ and allowed to incubate at 36°C for 1–4 hours before recording. To isolate miniature GABAergic postsynaptic currents (mGPSCs), 10 μM cyanquixaline (CNQX) and 0.5 μM tetrodotoxin (TTX) was added to the recording solution. All experiments were done at 32°C with a 1–2 ml/min flow rate. Before recording, brain slices were visualized under epifluorescence to verify the successful transduction of AAV‐Cre‐GFP in the SCN. For whole‐cell patch‐clamp recording, slices were imaged under infrared differential interference contrast optics and single cells were targeted from the dorsomedial SCN with borosilicate glass pipettes (4–7 MΩ) filled with (in mM): 145 KCl, 10 HEPES, 4 Mg‐ATP, 2 NaCl and 1 EGTA. A high concentration of Cl^−^ was used to facilitate the detection of mGPSCs from noise. Following gigaseal formation, the cell membrane was ruptured and allowed to equilibrate for 5 minutes before the onset of mGPSC recording. Whole‐cell recordings were performed with an Axopatch‐1D amplifier, filtered at 2 kHz, digitized at 5 kHz and acquired with Patchmaster v5.3. Cells were voltage‐clamped at −60 mV, and cells with holding currents >100 pA were excluded from the analysis. A small voltage step was used to monitor series resistance. Only cells with a stable series resistance were kept for mGPSC analysis.

### 2.9 mGPSC statistical analysis

The mGPSC frequency and amplitude data were compared using a generalized estimating equation (GEE) because not all measurements were independent: multiple mGPSCs were recorded per neuron, and multiple neurons were derived from the same mouse. The GEE allows us to account for the clustering of multiple measures from within the same mouse. This is essential to compute the correct standard errors to evaluate the estimated significance of any differences.

### 2.10 Wheel running locomotor activity recordings

Adult mice were housed individually in cages equipped with a running wheel and maintained in climate‐controlled environmental chambers. Housing light intensity was approximately 0.35 Wm^2^ (~300 lx). Wheel rotations were monitored through magnetic induction and acquired every 5 minutes with Vitalview software. Time‐series data were imported into Clocklab (Actimetrics, Wilmette, IL) for analysis of behavioural rhythmicity. Actograms were double‐plotted, and the display setting was set to ‘scaled.’ Chi‐square periodograms were generated from the last 7 days of wheel‐running data in constant darkness with a 1 min block size. The largest peak in the periodogram in the range of 12–36 hours was selected as the circadian period. The amplitude of this peak was used in the subsequent analysis as a proxy for the degree of circadian rhythmicity in individual animals. The *Vgat*‐depleted group (n = 16) includes *Vgat* ^lox/lox^;PER2::LUC mice injected with AAV‐Cre‐GFP (n = 11) in addition to *Vgat* ^lox/lox^ injected with AAV‐Cre‐GFP (n = 5). The *Vgat*‐intact group includes *Vgat* ^lox/wt^;PER2::LUC mice injected with AAV‐GFP mice (n = 2), *Vgat* ^wt/wt^ mice injected with AAV‐Cre‐GFP (n = 1) and *Vgat* ^wt/wt^ mice that did not receive an intracranial injection (n = 2).

### 2.11 Luminometry

Using sterile techniques, brains were quickly removed and submerged in chilled Hanks' buffered salt solution (HBSS). Briefly, the brain was blocked, and 300 μm thick coronal slices were prepared with a vibratome. SCN slices were trimmed to remove surrounding hypothalamic tissue and positioned onto Millicell Organotypic Cell Culture inserts (EMD Millipore, Darmstadt, Germany). Inserts were placed in 35 mm culture dishes containing 1.2 ml culture medium and sealed with vacuum grease. The culture medium consisted of DMEM supplemented with B27, 20 mM glucose, 10 mM HEPES, 4.2 mM NaHCO_3_, 25 μg/ml penicillin, 25 μg/ml streptomycin and 0.1 mM beetle luciferin. Dishes were placed into an Actimetrics Lumicycle, and photons were counted for 75 seconds every 10 minutes. Data were imported into the Actimetrics Lumicycle Analysis program (Actimetrics, Wilmette, IL). For each selection of data, the baseline was detrended by subtracting the 24‐hour and 3 hour running averages. The data was exported for subsequent analysis.

*2.12. Per1‐*Venus *imaging*

#### 2.11.1 Tissue preparation

Injected and uninjected male *Per1*‐Venus and *Vgat* ^lox/lox^;*Per1*‐Venus mice were housed under a 12:12 LD schedule. During the light phase (at approximately Zeitgeber time [ZT] 9 to 11), 5–20 week‐old mice were anaesthetized with isoflurane (Novaplus, UK) and euthanized consistent with the American Veterinary Medical Association Guidelines for the Euthanasia of Animals 2013. The brain was rapidly removed and placed in a sterile filtered (0.2 μm), ice‐cold slicing solution consisting of 1x HBSS (10x HBSS without Ca^2+^, Mg^2+^ and NaHCO_3_, Life Technologies, #14065); NaCO_3_ 4.5 mM, HEPES 10 mM, Penicillin/Streptomycin (100 U/ml each). Coronal hypothalamic slices (125–225 μm) containing the SCN were cut with a vibrating blade microtome (Leica‐Microsystems VT1000S; Wetzlar, Germany). The slices were placed on Millicell Organotypic Cell Culture inserts, and the insert was placed into a glass‐bottom 35 mm culture dish (MatTek Corp, Ashland, MA) containing 1.2 ml of a culture media. The culture media consisted of low glucose DMEM (Sigma, St. Louis, MO, # D2902) supplemented with B27 (50x serum‐free, Life Technologies # 17504–044), 19 mM glucose, 10 mM HEPES, 4.2 mM NaHCO_3_, 25 μg/ml penicillin and 25 μg/ml streptomycin. The media's pH was 7.2, and the osmolality was 295–310 mOsm/kg. The dish was sealed with sterile vacuum grease and placed in a 36°C temperature‐controlled chamber (ibidi GmbH, Martinsried, Germany) located on the stage of a fluorescent microscope (model Eclipse TE2000‐U, Nikon Corp, Japan) before the onset of the dark phase.

#### 2.11.2 Imaging

Venus fluorescence measurements were obtained by excitation at 500/20 nm with a 515 nm long pass dichroic and a 535/30 nm emission filter (Chroma Technology, Bellows Falls, VT, USA). Excitation light was supplied via a Lambda LS (Sutter Instrument, Novato, CA, USA) comprising a Xenon Arc Lamp and filter wheel. The light passed through a Nikon Eclipse TE2000 microscope using a 10x/0.3 Plan Fluor or a 20x/0.75 S Fluor Nikon objective. Images were captured every 20 minutes (600 ms duration exposure) using a cooled charge‐coupled device camera (CCD, Evolve 512 EMCCD, 16‐bit camera, Photometrics, Tucson, AZ, USA; or an ORCA‐ER CCD 12‐bit level camera; Hamamatsu, Japan) and controlled via the digital imaging software Metafluor (Molecular Devices, Sunnyvale, CA, USA).

#### 2.11.3 Imaging data analysis

Consecutive images from each trial were compiled into .gif files using ImageJ (ImageJ v1.48, U. S. National Institutes of Health, Bethesda, Maryland, USA). The “rigid body” option in the ImageJ Stackreg command is used to compensate for stage or slice drift that occurs during the imaging session. The background was subtracted using the ImageJ Subtract Background for each image using a sliding paraboloid radius of 20 to 50 pixels and subsequently subtracted from the corresponding cell fluorescence determined from each frame. Subsequent analysis was performed in Python using source code developed by Benjamin B. Bales (University of California, Santa Barbara) and John H. Abel (Harvard University) (Abel et al., 2016). The code is available at: https://github.com/bbbales2/neuron_tracker. Image files were analysed to track each cell's fluorescence intensity and position in the XY plane. Fluorescent cells located in the third ventricle were removed from the analysis. The software identifies neurons in each image, then connects the neurons between images in a timeseries. Intensity data were smoothed with a three‐frame rolling mean to minimize the effect of artefacts. To determine the classification of an individual cell, we performed linear fits in parallel to sine fits. We concluded that the cell was rhythmic if the error of the linear fit was larger than the error of the sine fit. Rhythmicity and synchrony were calculated via a Python algorithm, where each cell's fluorescence intensity was fit with an amplitude‐adjusted sine curve (scipy.optimize.curve_fit) for which the period was constrained to be between 23 and 25 hours. Synchrony was calculated by measuring the timing of peak fluorescence intensity within a 24‐hour cyclic period. Circular statistics were performed in the R programming language. A Rao's Spacing Test of Uniformity was done to establish validity. Then, if valid, a Rayleigh analysis was performed using the R package “Circular” written by Ulric Lund and Claudio Agostinelli (cran.r‐project.org/web/packages/circular/index.html, Jammalamadaka et al., 2001).

Subsequent analysis was performed using Excel (v14.4, Microsoft, Redmond, WA, USA), Igor (v6.3, Wavemetrics, Lake Oswego, OR, USA) and R (v3.02, R Development Core Team [2013]. R: A language and environment for statistical computing, R Foundation for Statistical Computing, Vienna, Austria. URL http://www.R-project.org).

## 3 RESULTS

### 3.1 Genetic disruption of *Vgat* attenuates intra‐SCN GABAergic neurotransmission

**FIGURE 1:**
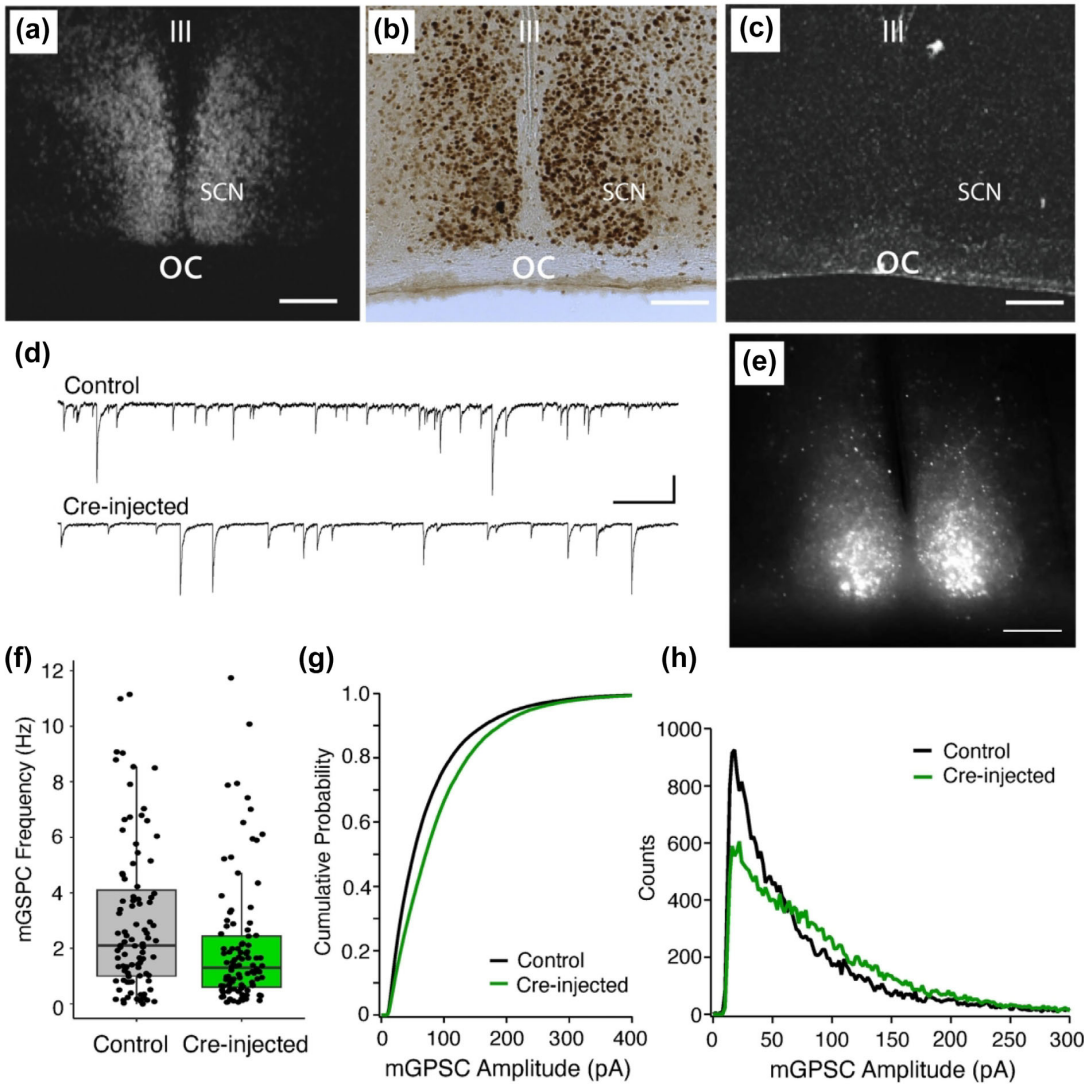
AAV‐Cre mediated disruption of GABAergic transmission in the *Vgat* ^lox/lox^ mouse SCN. (A) Isotopic in situ hybridization for *Vgat* in the SCN of an uninjected *Vgat* ^lox/lox^ mouse. (B) Cre immunostaining following bilateral injections of AAV‐Cre into the SCN of a *Vgat* ^lox/lox^ mouse. (C) Absence of *Vgat* mRNA expression in the SCN of the mouse shown in B. (D) Representative 5‐second traces of miniature GABA‐mediated post‐synaptic (mGPSCs) recorded from control (top trace) and the AAV‐injected SCN. The scale bar is 100 pA vertical and 0.5 sec horizontal. (E) Fluorescent (GFP) image of the SCN from a mouse injected with AAV‐Cre‐GFP and shown in D (bottom trace). (F) The frequency of mGPSCs was reduced by 37% in Cre‐injected SCN (8.1 vs 5.1 Hz; p < 0.05). The total number of events for each group was matched for comparison. (G) Cumulative probability plot showing that the average mGPSC amplitude was slightly increased (23.4%; p < 0.05) in AAV‐injected SCN (green line) compared to uninjected SCN (black line). (H) mGPSC event amplitude distribution for Cre‐injected (green line) and uninjected SCN (black line). Electrophysiological recordings were obtained from 12 AAV‐injected (100 neurons) and 12 uninjected *Vgat* ^lox/lox^ mice (98 neurons). OC = optic chiasm; SCN = suprachiasmatic nucleus; III = third ventricle.

Virtually all SCN neurons express *Vgat*, whose protein product VGAT is required to fill synaptic vesicles with GABA (Figure 1A; Weaver et al., 2018). To examine the role of GABA transmission in SCN network function in vivo we used transgenic mice in which *Vgat* is modified to contain loxP sites in the introns flanking exon 2 (*Vgat* ^lox/lox^), which contains the gene's translational start site (Tong et al., 2008) and delivered Cre‐recombinase to the SCN via viral vector (AAV‐Cre) injections. In transduced neurons, Cre‐mediated recombination deletes *Vgat*, leaving the neurons unable to load GABA into synaptic vesicles and release GABA synaptically (Tong et al., 2008). We injected AAV‐Cre into the SCN of adult male *Vgat* ^lox/lox^ mice (Figure 1B). Bilateral transduction of SCN neurons with AAV‐Cre produced a profound reduction in *Vgat* mRNA in the SCN and occasionally produced *Vgat* knockdown in the adjacent SPZ (Figure 1C; Extended Data Figure 1).

To quantify the loss of GABAergic input within the SCN, we recorded mGPSCs from SCN slices using whole‐cell patch clamp electrophysiology. Recordings were targeted to the dorsomedial SCN during ZT4 to ZT10, when the frequency of synaptic GABA activity in dorsomedial neurons is known to be constant (Itri et al., 2004). Local VGAT depletion decreased the frequency of mGPSCs by 37% (from 8.1 to 5.1 Hz; p < 0.05 using GEE, 100 neurons recorded from 12 Cre‐injected and 98 neurons from 12 uninjected *Vgat* ^lox/lox^ mice, Figure 1D‐F). A small increase in the mean mGPSC amplitude was also observed (+23.4%, p < 0.05 using GEE, Figure 1G, H). Closer examination of the mGPSC amplitude distribution revealed that, compared to uninjected controls, SCN neurons from AAV‐Cre injected *Vgat* ^lox/lox^ mice exhibited a reduction in the proportion of small amplitude mGPSCs (less than 75 pA), most likely representing loss of intra‐SCN transmission, and a concomitant increase in the proportion of medium amplitude mGPSCs (75 to 200 pA, Figure 1H) which we hypothesize represents preservation of GABAergic afferents from other areas of the brain (Card & Moore, 1982, 1989; Hanna et al., 2017; Moore & Card, 1994) or potentially even from the retina via the retinohypothalamic tract (Sonoda et al., 2020). Collectively, these in vitro findings confirm that our manipulation disrupts synaptic GABA transmission within the SCN neural network.

### 3.2 Bilateral depletion of GABAergic transmission from SCN neurons causes loss of physiological rhythmicity in constant darkness

**FIGURE 2:**
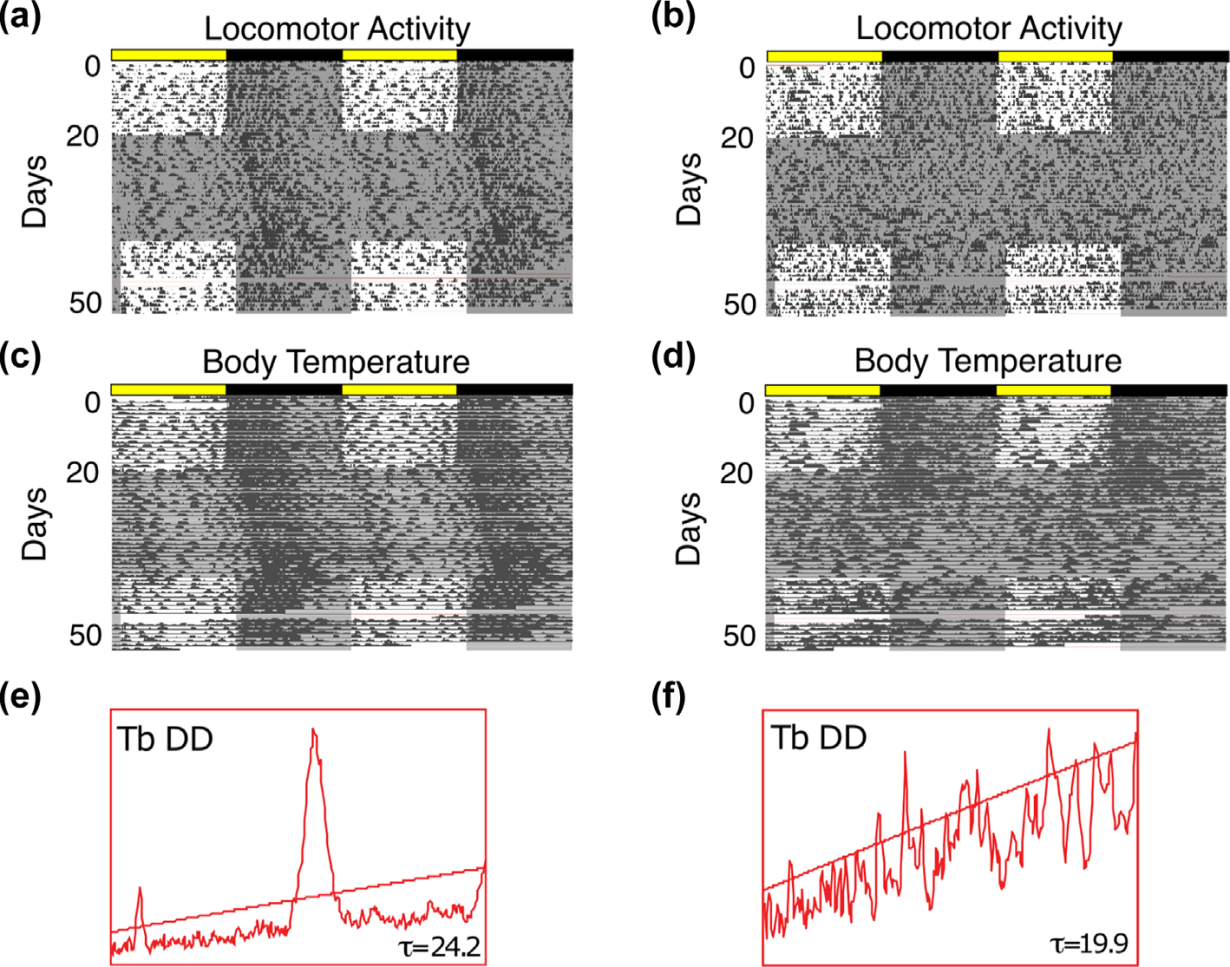
Deletion of *Vgat* in SCN neurons produced behavioural and physiological arrhythmicity in vivo. (A, C) Double‐plotted LMA (A) and Tb (C) actograms from a mouse showing entrainment to a 12:12 LD cycle and a high amplitude, sustained free‐running rhythm in constant darkness (DD is shown by darker shading). (B, D) Double‐plotted LMA (B) and Tb (D) actograms of a mouse show entrainment to a 12:12 LD cycle but progression to arrhythmia in DD. (E, F) Periodogram analysis of the Tb data from the last 7 days of DD shows the robust 24 h rhythm component in the control mouse (E; data from C) compared to the loss of the 24 h rhythm in the AAV‐Cre injected mouse (F; data from D). The location and extent of the bilateral Cre injections were confirmed histologically.

To assess the behavioural consequences of VGAT depletion in the SCN, we used telemetric devices to monitor body temperature (Tb) and locomotor activity (LMA) in *Vgat* ^lox/lox^ mice injected with AAV‐Cre (n = 11), a control vector expressing GFP (AAV‐GFP) (n = 6) and in uninjected (n = 10) *Vgat* ^lox/lox^ littermates. To provide sufficient time for recovery, Cre‐mediated recombination and subsequent VGAT depletion to occur, we began Tb and LMA recordings 2–3 weeks following surgery (Kaur et al., 2013; Pedersen et al., 2017). During a 12:12 light–dark (LD) cycle, all mice demonstrated high amplitude diurnal Tb (Figure 2C, D) and LMA rhythms (Figure 2A, B). The mice were subsequently released into constant darkness (DD) to assess the circadian rhythmicity of Tb and LMA. Control mice exhibited a consolidated free‐running rhythm in DD (Figure 2A, C, E, Extended Data Figure 1), while *Vgat* ^lox/lox^ mice with bilateral Cre injections into the SCN (n = 6; confirmed histologically) showed a reduction of the Tb and LMA circadian rhythm amplitude (Figure 2B, D, F and Table 1).

The SPZ is located approximately 400 μm dorsal to the SCN and injections targeting the SCN sometimes produce off‐target Cre expression in the SPZ (Extended Data Figures 1 & 2). SPZ neurons receive synaptic input from the SCN, and may be involved in relaying rhythmicity from the SCN (Chou et al., 2003; Lu et al., 2001; Todd et al., 2018). Therefore, to confirm that VGAT depletion from the SCN is necessary for the observed behavioural arrhythmicity, we analysed Tb rhythms (Extended Data Figure 2) from *Vgat* ^lox/lox^ littermates with either AAV‐Cre (n = 9; Extended Data Figure 2A, C) or AAV‐GFP (n = 9; Extended Data Figure 2B, D) targeted to the SPZ. Because our intracranial injections approach the SCN dorsally, these mice lack SCN transduction. During either a 12:12 light–dark cycle or constant darkness, the mice with bilateral AAV‐Cre in the SPZ remained strongly rhythmic (Extended Data Figures 1B, C; Extended Data Figures 2D‐F; Table 2). Therefore, the results of SPZ VGAT depletion demonstrate that the observed behavioural disruption is not due to alterations of GABA transmission in the SPZ (Extended Data Figure 2).

We next sought to determine whether removing the LD cycle from the time of AAV‐Cre injection was able to reveal arrhythmicity. A second cohort of *Vgat* ^lox/lox^ mice (n = 10) with bilateral injections of AAV‐Cre into the SCN were generated and placed directly into DD following post‐surgical recovery. In this group, all five of the mice that showed bilateral Cre transduction of SCN neurons became arrhythmic within 3–4 weeks following surgery, while none of the five mice that retained circadian rhythms had bilateral depletion of VGAT in the SCN (Table 1; Extended Data Figure 2E, F). This loss of rhythmicity in constant darkness suggests that the rhythmic behaviour observed under LD is not circadian in nature but imposed by the acute effects of light (light masking).

### 3.3 Effect of VGAT depletion on PER2 clock gene rhythm in the SCN

**FIGURE 3:**
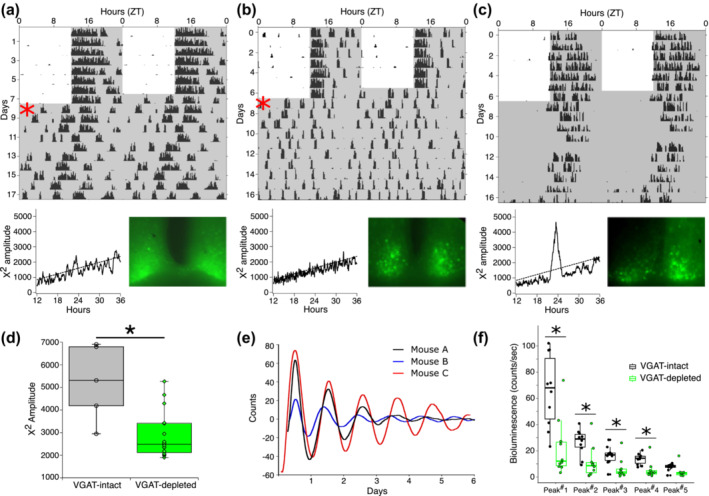
PER2 rhythmicity persists but is reduced in amplitude in VGAT‐depleted mice. (A‐C) Wheel running activity of *Vgat* ^lox/lox^;PER2::LUC mice injected with AAV‐Cre (upper panels). The corresponding *X* ^2^ periodograms and epifluorescent images of AAV‐Cre SCN transduction are shown below each actogram. In A and B, note the rapid change of activity onset in DD (* in actogram), suggesting that the diurnal rhythmicity observed in LD may be attributed to negative light masking. (D) Summary data for the wheel‐running activity in DD shows that the mean *X* ^2^ amplitude was significantly lower in AAV‐Cre injected VGAT‐depleted mice (n = 16) than in VGAT‐intact control mice (n = 5, p = 0.0106 two‐tailed *t*‐test), indicative of compromised rhythmicity. (E) SCN slices from AAV‐Cre injected *Vgat* ^lox/lox^;PER2::LUC mice demonstrated PER2 bioluminescent rhythmicity in culture. Traces shown in (E) are derived from the mice of A‐C. (F) Summary data for the PER2 bioluminescence traces show that the amplitude of the bioluminescence signal was significantly lower in AAV‐Cre injected *Vgat* ^lox/lox^;PER2::LUC mice than control mice at each of the first four peaks (n = 10 for each group, generalized estimating equation (GEE), * p < 0.01).

To determine whether SCN rhythmicity is disrupted in the arrhythmic mice, we recorded molecular rhythms within the SCN itself using double transgenic *Vgat* ^lox/lox^;PERIOD2::LUCIFERASE (PER2::LUC) mice. Before bioluminescent recording, wheel‐running activity was monitored under a 12:12 LD cycle for 10 days. The wheel‐running activity of VGAT‐depleted mice (n = 16) was compared to VGAT‐intact mice (n = 5). In accordance with the telemetry data, VGAT‐depleted mice demonstrated diurnal wheel‐running activity under LD but circadian disruption and arrhythmia under constant darkness (Figure 3A, B & D). The average *X* ^2^ periodogram amplitude was 5229 ± 1699 for the VGAT‐intact mice (n = 5), indicating normal and robust circadian rhythmicity. Although there was some variability in the extent of behavioural disruption apparently reflecting the extent of bilateral Cre expression (Figure 3C), the average *X* ^2^ periodogram amplitude was significantly smaller in the VGAT‐depleted mice, indicating a reduction in rhythm strength (Figure 3D; average amplitude 2902 ± 1034; n = 16; *t*‐test, p = 0.001).

After recording wheel‐running activity in the *Vgat* ^lox/lox^;PER2::LUC mice, organotypic SCN slices were prepared to measure molecular clock rhythms in vitro. Surprisingly, despite disrupted wheel‐running rhythmicity, behaviourally arrhythmic mice still demonstrated rhythmic PER2 expression within the SCN (Figure 3E). Using the rate of damping as an index of the degree of synchrony between neurons (Maywood et al., 2011; Mieda et al., 2015) and after normalizing the amplitude of each trace to the first bioluminescence peak, we found no difference in the amplitude of the third peak between VGAT‐depleted (0.36 ± 0.07, n = 10) and VGAT‐intact mice (0.28 ± 0.05, n = 10), (p = 0.62, two‐tailed Student's *t*‐test). Furthermore, we found no difference in period for the cycles of PER2 bioluminescence between groups (24.82 ± 0.43 hrs for VGAT‐depleted (n = 10) v. 24.28 ± 0.26 hrs for VGAT‐intact mice (n = 10), p = 0.38, two‐tailed Student's *t*‐test). However, the absolute amplitude of the first 24‐hour peak was significantly reduced in the Cre‐injected mice (30.45 ± 7.45 counts/s, two‐tailed *t*‐test, p < 0.002, n = 10) when compared to control mice (65.59 ± 8.73 counts/s, n = 10), and this difference in absolute amplitude persisted for the 4 subsequent days in culture (Figure 3F). Thus, although PER2 rhythmicity persists in the SCN, the signal's amplitude is diminished, which may underlie the disruption of overt behavioural rhythmicity.

### 3.4 Effect of VGAT depletion on *Per1* clock gene rhythm and synchronicity between individual SCN neurons

**FIGURE 4:**
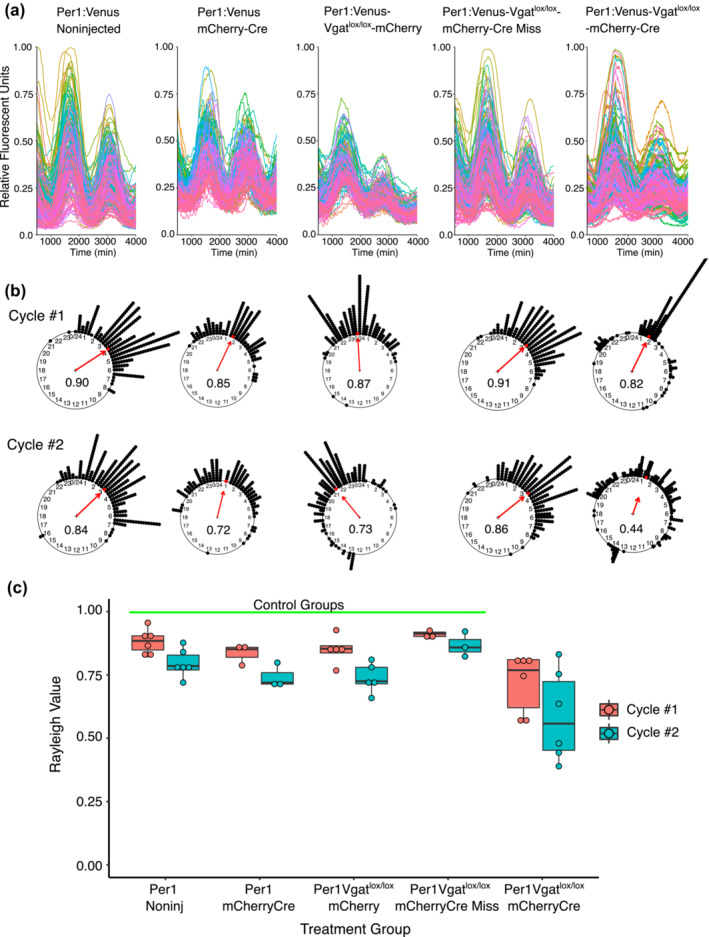
Time‐lapse examination of slice explants following AAV‐Cre injection into the SCN of *Vgat* ^lox/lox^ *;Per1‐*Venus mice reveals reduced synchrony between SCN neurons. (A) Individual SCN cell recordings of *Per1* expression in SCN slices from uninjected *Per1‐*Venus*, Per1‐*Venus mice injected with AAV‐Cre‐mCherry*, Vgat* ^lox/lox^ *;Per1‐*Venus mice injected with AAV‐mCherry, and *Vgat* ^lox/lox^ *;Per1‐*Venus mice injected with AAV‐Cre‐mCherry. (B) Rayleigh plots of the slices shown in (A) after 24 (Cycle #1) and 48 (Cycle #2) hours in culture. (C) Summary boxplot of the Rayleigh values calculated from each group during the two cycles.

To examine the molecular clock rhythms in the SCN of VGAT‐depleted mice at a cellular level, we visualized individual SCN neurons in slice cultures using time‐lapse recordings from *Per1*‐Venus mice, in which the *Per1* promoter drives expression of the fluorescent protein Venus (Cheng et al., 2009). For our recordings, *Vgat* ^lox/lox^;*Per1*‐Venus mice (n = 9) received SCN‐directed injections of AAV‐mCherry‐Cre, while uninjected *Per1‐*Venus mice (n = 6), *Per1‐*Venus mice injected with AAV‐Cre‐mCherry (n = 3) and *Vgat* ^lox/lox^;*Per1‐*Venus mice injected with AAV‐mCherry (n = 5) served as controls (Figure 4). Successful SCN injections were identified by the expression of mCherry. To measure synchrony between cells, we constructed Rayleigh plots to monitor the time of peak fluorescence of individual cells, across 24‐hour cycles. Control slices from uninjected animals had a mean first peak Rayleigh coefficient of 0.88 ± 0.05 (mean ± SD, n = 6 slices, Figure 4) and second peak Rayleigh coefficient of 0.80 ± 0.06 (n = 6 slices, Figure 4) consistent with previously reported values for rhythmic SCN slices (Brancaccio et al., 2013; Freeman et al., 2013; Herzog et al., 2015). AAV‐Cre‐mCherry injected *Per1‐*Venus mice had a very similar mean Rayleigh coefficient of 0.84 ± 0.04 on the first day and a value of 0.74 ± 0.05 on the second day (n = 3 slices), as did *Vgat* ^lox/lox^;*Per1‐*Venus mice injected with AAV‐mCherry, which had a mean first Rayleigh coefficient of 0.85 ± 0.06 and 0.71 ± 0.1 during the second peak (n = 5). However, the *Vgat* ^lox/lox^;*Per1‐*Venus mice injected with AAV‐Cre‐mCherry, where Cre recombination produced VGAT depletion in the SCN, had a mean Rayleigh coefficient of 0.73 ± 0.13 on the first day and a mean value of 0.59 ± 0.18 on the second day (n = 6). In three experiments from *Vgat* ^lox/lox^;*Per1‐*Venus mice injected with AAV‐Cre‐mCherry in which the injection missed the SCN, the mean Rayleigh coefficient was 0.91 ± 0.02 on the first day and 0.87 ± 0.05 on the second day (n = 3). When comparing across groups, the mean Rayleigh coefficient for the *Vgat* ^lox/lox^;*Per1‐*Venus mice injected with AAV‐Cre‐mCherry was significantly smaller than any of the four control groups (Cycle 1 ‐ Kruskal‐Wallis x^2^ = 12.38, df = 4, p = 0.015; Day 2 ‐ Kruskal‐Wallis x^2^ = 10.391, df = 4, p = 0.034; Tukey–Kramer‐Memenyi all pairs test with a Bonferroni correction, Figure 4). These data reveal that, although many SCN neurons continue to display low‐amplitude oscillations after VGAT‐depletion, disruption of SCN GABAergic neurotransmission compromises synchrony between neurons, resulting in an insufficient SCN output signal to sustain behavioural rhythms.

## 4 DISCUSSION

Here we show that acute depletion of VGAT in the SCN, and therefore disruption of synaptic GABA release from SCN neurons, compromises coherent circadian rhythmicity in adult mice. VGAT‐depleted mice retained diurnal rhythms of LMA, Tb and wheel running activity in LD, but became arrhythmic in constant darkness. In VGAT‐depleted mice, the SCN neural network was also disrupted: the amplitude of SCN PER2::LUC bioluminescence was reduced, and the phase of molecular rhythms across SCN neurons was desynchronized. Together these data suggest that in the absence of daily light input, local GABAergic transmission is critical for maintaining an SCN network with sufficient synchrony to drive coherent behavioural and physiological rhythms in vivo.

Remarkably, GABA disruption demonstrated a more profound phenotype than the deletion of certain clock genes. Rhythmicity is compromised but remains intact in *Clock* ^*−/−*^, *Cry1* ^−/−^, *Cry2* ^−/−^, *Per1* ^−/−^, *Per3* ^−/−^, *Rev‐erb*α^−/−^, *Ror*α^−/−^ and *Ror*β^−/−^ mice. Double knockouts of these clock genes are often required to induce arrhythmicity (Lowrey & Takahashi, 2011). Therefore, the severity of circadian disruption observed after VGAT depletion underscores the importance of GABA transmission for SCN function.

A few technical points should be considered when interpreting our data. First, *Vgat* deletion depends on the transduction of neurons and subsequent expression of Cre recombinase, and it is likely that not all SCN neurons were transduced in any single injection. In our attempts to delete *Vgat* expression in mice where we recorded biological rhythmicity, only 6 of 15 had verified complete deletion of *Vgat* gene expression bilaterally in the SCN, and all of those mice demonstrated loss of circadian rhythmicity in DD. Deletion of *Vgat* is expected to disrupt a neuron's ability to release GABA synaptically, but not to directly affect the cycling of the molecular clock within an individual neuron. Indeed, our *Per1*‐Venus recordings indicate that many Cre + SCN neurons continued to show cycling despite reduced synchrony within the neural network. Previous studies have observed that only about 50–60% of SCN neurons remain rhythmic in low‐density neuronal cultures where there is limited interneuronal communication (Webb et al., 2009, 2012), suggesting that two populations of SCN neurons exist, where one is dependent on the other for rhythmic input. Our results support this idea by showing that if synchrony is disrupted in a threshold percentage of SCN neurons, the remaining network is insufficient to produce behavioural rhythmicity during DD. Indeed, the loss of circadian rhythmicity observed in our work recapitulates the loss of rhythmicity observed following inhibition of synaptic transmission from NMS + neurons (Lee et al., 2015).

While GABA is a critical neurotransmitter for intercellular SCN communication, it is also found in SCN efferent projections, e.g., those to the SPZ, preoptic area and retrochiasmatic nucleus (Morin & Allen, 2006). Therefore, it is possible that these (or other) efferent nuclei require GABAergic input from the SCN to orchestrate rhythmicity, and that the behavioural arrhythmicity we observed reflects a disruption in this signal. However, under LD our animals showed normal locomotor and body temperature rhythms after VGAT depletion. The animals exhibited arrhythmic behaviour only after being placed in constant darkness, which argues against deficits in the efficacy of efferent projections to other nuclei, and for a common defect in the circadian pacemaker.

Even without GABAergic efferents, a functional SCN could use peptides or other molecules (e.g., transforming growth factor‐α) to maintain circadian rhythmicity (Kramer et al., 2001; Silver et al., 1996). Indeed, previous studies have established that paracrine factors are sufficient for a transplanted SCN to drive some behavioural rhythmicity (Ralph et al., 1990; Silver et al., 1996). Our PER2 and *Per1* imaging data demonstrate that even if downstream effects contribute to the loss of behavioural rhythmicity, there are still direct effects on the circadian clock in the SCN following a reduction in SCN GABA transmission.

Despite the behavioural fragmentation observed in VGAT‐depleted mice, the PER2::LUC signal remained partially rhythmic. This result is in accordance with PER2::LUC luminometry from embryonic *Vgat* ^*−/−*^ SCN tissue (Ono et al., 2019). Still, our PER2::LUC experiments may have underestimated the extent to which the SCN network was disrupted in vivo. Dissection‐induced resetting may have occurred during slice preparation. Indeed, dissection‐induced resetting has been previously reported in luciferase models that are arrhythmic before dissection. However, our *Per1*‐Venus imaging experiments, which allow for a more direct assessment of individual neurons, support the conclusion that intercellular GABAergic signalling is required for synchrony in the SCN neural network. Though they are homologs with a similar phase relationship, important distinctions have been made between PER1 and PER2. Some SCN neurons express both PER1 and PER2, but others express only one or the other (Cheng et al., 2009; Riddle et al., 2017). PER1 and PER2 demonstrated differential induction and localization in the SCN after mice were subjected to phase‐advancing and phase‐delaying light pulses, suggesting that PER1 and PER2 have different influences on the molecular clock (Yan & Silver, 2002). Furthermore, microarray analysis revealed that PER1 and PER2 regulate the expression of separate sets of clock‐controlled genes (Zheng et al., 2001). We observed a reduction in PER2 expression but could only resolve single‐cell rhythms of *Per1*‐Venus. Therefore, additional studies will be necessary to determine if GABA transmission selectively disrupts PER1 intracellular signalling.

It has become increasingly clear that the SCN neural network is essential to maintain synchrony between its autonomous cellular oscillators and produce a robust and reliable circadian output (Mohawk & Takahashi, 2011). While GABAergic neurotransmission has been proposed to play a role in the synchronization of SCN neurons, this function has remained controversial (Aton et al., 2006; Liu & Reppert, 2000). For example, Freeman et al. (2013) concluded that GABA_A_ activation actually elicits desynchrony in the SCN, whereas a modelling study by DeWoskin et al. (2015) suggested that excitatory GABA transmission increases synchrony between SCN neurons while inhibitory GABA transmission decreases synchrony (DeWoskin et al., 2015). GABA can contribute to circadian clock function by refining the frequency and regularity of action potential firing of individual SCN neurons. Both synaptic and extrasynaptic receptors mediate neurotransmission in the SCN, and both synaptic and a GABA_tonic_ show diurnal rhythms (Moldavan et al., 2015, 2017, 2021). Neurotransmission mediated by GABA_A_ receptors containing γ2 or δ subunits is essential in maintaining neuronal synchrony in the SCN and the amplitude of the daily activity‐rest cycles. Modulating the expression level of GABA_A_ receptors containing these subunits may be one mechanism regulating the strength of coupling between SCN neurons (Granados‐Fuentes et al., 2024). GABA_A_ receptors containing γ2 subunits are generally located at synapses. In contrast, δ subunits are expressed in extrasynaptic GABA_A_ receptors, contributing to the observed tonic GABA_A_ receptor‐mediated current (GABA_tonic_) in SCN neurons (Moldavan et al., 2021). An SCN neural network computational model proposed that GABA_tonic_ shifts circadian rhythms and contributes to oscillator synchrony. In contrast, the synaptic GABA_A_ currents do not significantly affect molecular timekeeping (DeWoskin et al., 2015; Myung et al., 2015). Extrasynaptic GABA_A_ receptors activated by extrasynaptic GABA play a role in circadian clock activity, and the circadian activity of GABA transporters regulates their activity (Moldavan et al., 2017; Patton et al., 2023). This intricate interplay of receptor subunits and their roles in GABAergic neurotransmission adds a layer of complexity to our understanding of the SCN neural network.

Developmental deletion of *Vgat* from SCN neurons that express the insulin‐2 promoter (RIP) is without effect on diurnal or circadian rhythms, although the percentage of SCN neurons that express RIP was not specified (Kong et al., 2012). A recent study has used a similar genetic approach to ours to produce VGAT depletion in the SCN (Ono et al., 2019). Notably, this study supports our conclusion that loss of VGAT in the SCN compromises behavioural rhythmicity, although the loss of rhythmicity in DD was not as complete as in our animals. However, the depletion of VGAT immunoreactivity was only 31% in the Ono et al. experiments (Ono et al., 2019). It is possible that the behavioural results from our animals with verified complete deletion of *Vgat* expression in the SCN indicated more complete deletions. This, coupled with the fact that while we routinely waited 2–3 weeks after Cre injection, Ono et al. began recordings immediately after transduction, could both have contributed to the discrepancies in the extent of arrhythmicity. Interestingly, two recent studies have shown that selective deletion of VGAT from either SCN AVP + (Maejima et al., 2021) or VIP + (Todd et al., 2020) cells alters the timing of the locomotor rhythm or influences the phase angle of entrainment of body temperature, respectively. Still, neither manipulation resulted in arrhythmicity. The loss of circadian rhythmicity observed in our work recapitulates the loss of rhythmicity observed following inhibition of synaptic transmission from NMS + neurons (Lee et al., 2015). NMS + neurons constitute approximately 40% of all SCN neurons and include both AVP + and VIP + neurons. Selective VGAT deletion from SCN NMS + neurons reduced the amplitude but not the period of behavioural rhythms (Bussi et al., 2023). These data suggest that broader VGAT depletion in the SCN is required to produce the circadian disruption we observed.

Several studies have reported that VIP signalling (Aton et al., 2005), but not GABA (Aton et al., 2006), is necessary to maintain neuronal synchronization in SCN slices. Specifically, mice lacking VIP or the VIP receptor, VPAC2, show compromised behavioural rhythmicity (Aton et al., 2005; Colwell et al., 2003; Harmar et al., 2002). In vitro, SCN neurons from VPAC2 knockout mice demonstrate desynchrony and reduced amplitude *Per1*‐bioluminescence rhythms (Brown et al., 2007; Hughes et al., 2008; Maywood et al., 2006). However, subsets of these mice show no behavioural phenotype (Aton et al., 2005; Colwell et al., 2003; Harmar et al., 2002), suggesting that other non‐VIP mechanisms are involved in SCN neuronal synchronization. Our data suggest that we are targeting GABA transmission exclusively while leaving VIP transmission unperturbed (Extended Data Figure 3). Therefore, in conjunction with prior studies, our results contribute to a model in which both fast synaptic GABA transmission along with slower peptidergic neuromodulation are essential to produce SCN synchrony and behavioural rhythmicity (Evans et al., 2013).

## AUTHOR CONTRIBUTIONS

Conceptualization, H.S.G, N.K., C.N.A, W.D.T., C.B.S. and P.M.F.; Methodology, H.S.G, N.K., C.N.A, A.N.G, L.M.H., R.P.I., W.D.T., C.B.S. and P.M.F.; Investigation, H.S.G, N.K., C.N.A, O.C., A.N.G, L.M.H., R.P.I., W.D.T., C.B.S. and P.M.F Writing – Original Draft, H.S.G., N.K., C.N.A., C.B.S., P.M.F.; Writing – Review & Editing, H.S.G, N.K., C.N.A, O.C., A.N.G, L.M.H., R.P.I., W.D.T., C.B.S. and P.M.F; Funding Acquisition, C.N.A., C.B.S., P.M.F.; Resources, C.N.A., R.P.I., W.D.T., C.B.S., P.M.F.; Supervision, C.N.A., C.B.S., P.M.F.

## CONFLICT OF INTEREST STATEMENT

The authors have no conflicts of interest to declare.

### PEER REVIEW

The peer review history for this article is available at https://www.webofscience.com/api/gateway/wos/peer-review/10.1111/ejn.16582.

## Supporting information


**Data S1.** Supporting information.

## Data Availability

The data are available upon request.
