# Supplementary material for: GABAergic signalling in the suprachiasmatic nucleus is required for coherent circadian rhythmicity
Source: Eur J Neurosci. 2024 Nov 18;60(11):6652–67. doi: 10.1111/ejn.16582 (PMC11612841; doi:10.1111/ejn.16582)
Supplement: Supplementary file 1 — Data S1. Supporting information. [file EJN-60-6652-s001.pdf]

Extended Data

Extended Data Figure 1. Bilateral SCN injection and transduction of AAV-Cre in wildtype mice is without effect on behavioral or physiological rhythmicity. (A) Bilateral injections of AAV-Cre were placed into the SCN of wildtype (C57/BL6) mice. (B and C) Injected mice showed normal body temperature patterns (B) and locomotor activity (C) under both LD and DD conditions.

Extended Data Figure 2. Targeted injections of the SPZ do not affect circadian Tb or LMA rhythms. (A) Photomicrograph showing bilateral Cre transduction of the SCN with sparse but evident labeling in the dorsally-adjacent SPZ. (B) Photomicrograph showing bilateral Cre transduction in the ventral and dorsal SPZ, but not in the SCN. (C, D) Double-plotted Tb actograms showing unaltered Tb rhythms in LD and DD following SPZ-directed AAV-GFP (C) or AAV-Cre (D) injections into *Vgat*$^{lox/lox}$ mice. Scale bar = 200 μm. Abbreviations: oc – optic chiasm, 3V – third ventricle; SCN – suprachiasmatic nucleus, SPZ – subparaventricular zone. (E) A heatmap showing overlapping regions of Cre transduction in *Vgat*$^{lox/lox}$ mice (n = 9) with SCN-directed AAV-Cre injections that demonstrated arrythmicity in constant darkness. The extent of transduced somata and location were slightly different for each injection due to variability in both injection volumes (10 – 30 nl) and the location of the pipette tip used for the injection. Despite variable Cre transduction in areas of the hypothalamus dorsal and lateral to the SCN, the heatmap reveals that bilateral Cre transduction within the SCN was the common feature in arrhythmic animals. (F) Additional *Vgat*$^{lox/lox}$ mice (n = 5) with AAV-Cre SCN-directed (but mostly off-target) injections, individually mapped and overlaid, from mice that retained high amplitude rhythms in DD. Note that Cre did not transduce the SCN bilaterally in any of these rhythmic animals.

Extended Data Figure 3. Bilateral SCN injection and transduction of Cre-AAV results in deletion of *Vgat* but does not produce non-specific effects on SCN vasoactive intestinal polypeptide (VIP). (A) Bilateral injections of Cre-AAV were

placed into the SCN of *Vgat*$^{lox/lox}$ mice to determine if Cre-AAV might produce nonspecific reductions in another SCN signaling molecule, in this case, VIP. These mice were not used in the physiologic or behavioral recordings of the present study but were generated as a separate cohort for anatomical analysis. The mice were given ICV colchicine injections 36 hours before perfusion. (B) absence of *Vgat in situ* signal (cf. with panel E, which shows *Vgat* signal in SCN of the uninjected control mouse). (C) VIP immunohistochemistry in the colchicine-treated brains revealed comparable morphology and quantity of VIP neurons between (C) Cre-AAV treated and (F) uninjected *Vgat*$^{lox/lox}$ mice. (D-F) Corresponding sections from a littermate *Vgat*$^{lox/lox}$ mouse that did not receive injections of Cre-AAV. Brain sections from all mice were batch-processed for *Vgat* and VIP.

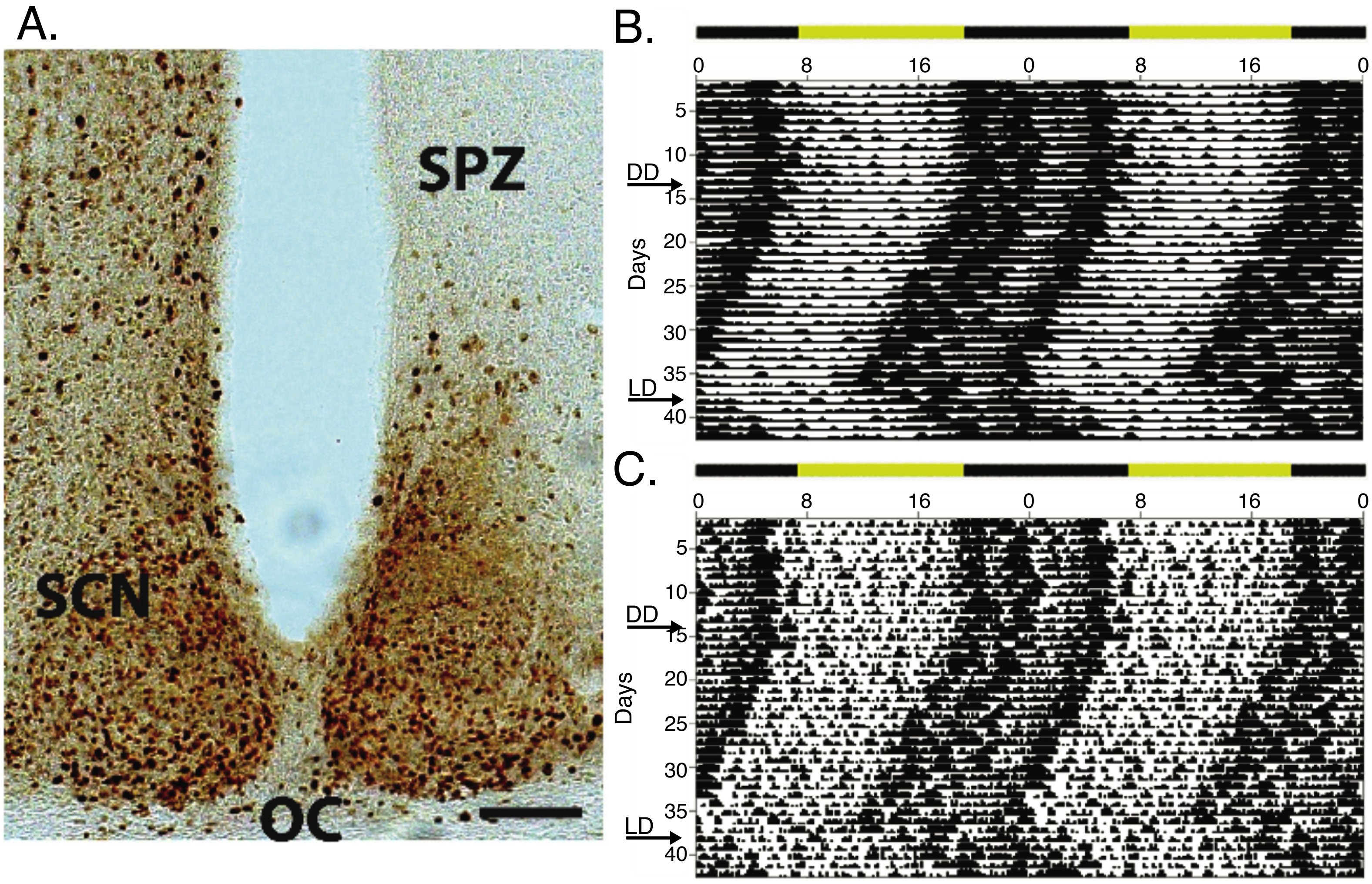

Extended Data Figure 1

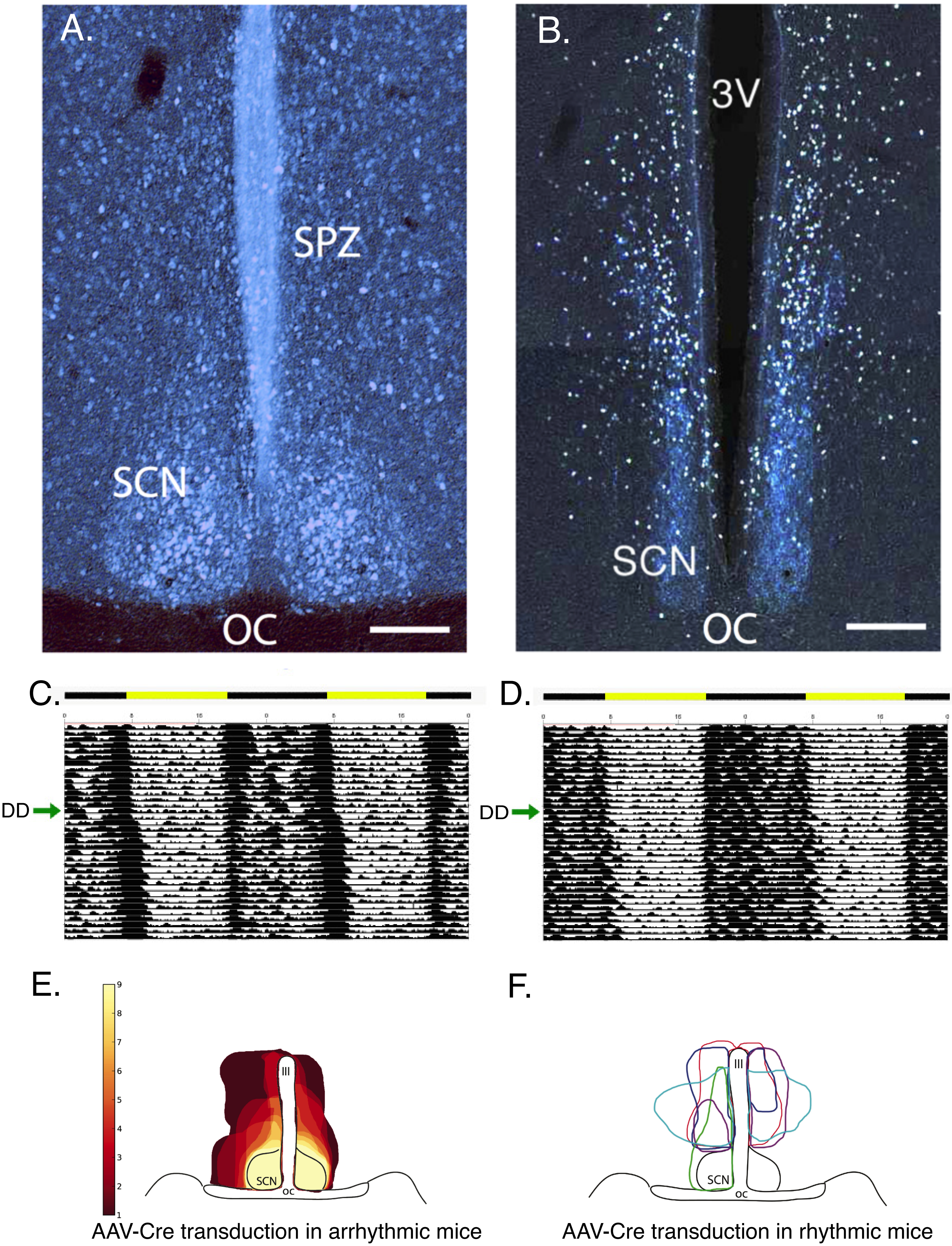

Extended Data Figure 2

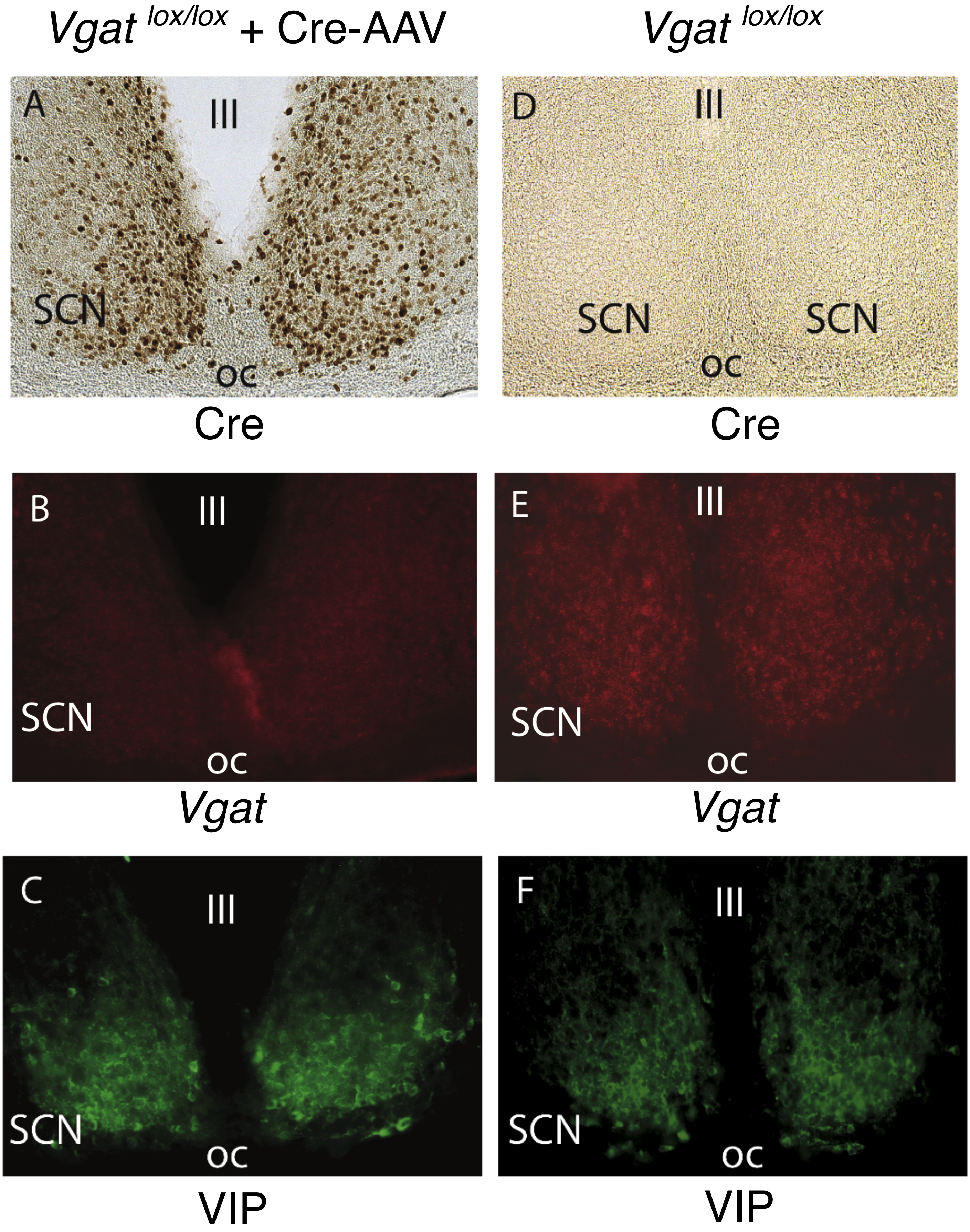

Extended Data Figure 3
